# Supplementary material for: Content-rich biological network constructed by mining PubMed abstracts
Source: BMC Bioinformatics. 2004 Oct 8;5:147. doi: 10.1186/1471-2105-5-147 (PMC528731; doi:10.1186/1471-2105-5-147)
Supplement: Additional File 2 — The original results of the above study (non-essential files are deleted to keep the file size under the limit set by BMC bioinformatics). [file 1471-2105-5-147-S2.bz2 › chilibotAdditionalFile2/dip30/69ID2966143E269/html/RECB_GAM.html]

 


 **RECB** and **GAM** 
  
Found 22 abstracts in PubMed, retrieved 22.  
 

 What does Google say? 
 PDF only 
| .edu only 

---

**Interactive relationship** (e.g. stimulation, inhibition, etc)

**Inhibitory relationship**- The lysogens overproducing the  **RecB**  subunit of RecBCD enzyme could titrate  **Gam**  protein and thus prevent the growth of T4 2.  Ref: 8335632 J Bacteriol, 1993
- It may be inferred that lambda  **Gam**  protein, which otherwise inhibits RecBCD DNase and thus permits the growth of this phage, is bound by the helicase domain of  **RecB** .  Ref: 11130867 Res Microbiol, 2000
- The  **recB**  C bacteria expressing gamS were completely or partially similar to recC mutants with respect to certain phenotypes defective plating of phages P1 and P2, ability to plate in a recA background lambda red  **gam**  phages, reduced resistance to UV irradiation, defective SOS induction, decreased colony forming ability.  Ref: 2943636 Gene, 1986
**Neutral relationship**- It is therefore concluded that the  **RecB**  subunit is capable of binding  **Gam**  protein.  Ref: 8335632 J Bacteriol, 1993
- **gam**  dependent radioresistance is observed in  **recB**  recC recD but not in  **recB**  recC recD cells.  Ref: 1655052 Biochimie, 1991
- **gam**  lambda carrying the same palindrome grow on both hosts and on a host deleted for the  **recB** , C and D genes.  Ref: 2531105 Genetics, 1989
